# Intrathoracic migration of a K-wire after percutaneous fixation of a proximal humerus fracture.

**DOI:** 10.1016/j.tcr.2021.100425

**Published:** 2021-02-10

**Authors:** A.J. van Hasselt, J.Th. Hooghof, M.R. Huizinga, J.J.A.M. van Raay

**Affiliations:** Department of Orthopaedic Surgery, Martini Hospital, Groningen, the Netherlands

**Keywords:** Migration of K-wire, Kirschner wire, Percutaneous fixation, Proximal humerus fracture

## Abstract

Proximal humerus fractures are common in elderly patients. Not all patient are fit for major surgery. Percutaneous fixation can be a suitable option though surgeons should be aware of the risks and complications. This case is about a 90-year-old woman with a proximal humerus fracture. After closed anatomical reduction we performed percutaneous K-wire fixation of the humerus fracture with a single K-wire. Five days postoperatively the patient experienced increased pain and dyspnea due to a pneumothorax caused by intrathoracic migration of the K-wire. Percutaneous fixation can be a suitable treatment for low-maintenance and fragile patients but surgeons should act with caution. Multiple threaded K-wires with a bend-free end should be used to reduce the risk for loss of repositioning or migration of the K-wire.

## Introduction/background

Fractures of the proximal humerus are common injuries and account for 5% of all fractures [1]. In the elderly population, proximal humerus fractures are the third most common osteoporotic fractures following the distal radius and femoral neck [2]. These fractures affect 80–85% of people older than 50, with a peak incidence between ages 60 and 90 [1,3]. Up to 80% of patients with proximal humerus fractures will not benefit from surgery and will regain acceptable-to-good function of the shoulder with conservative treatment [3,4]. Surgery should only be considered when the fracture is more complex or when shoulder function is seriously compromised [3]. Of all proximal humerus fractures, 49% are minimally displaced, 28% are surgical neck fractures and 9% are three-part greater tuberosity and surgical neck fractures [5]. Percutaneous Kirschner wire (K-wire) fixation used to be a common surgical technique for proximal humerus fractures [3,6,7]. In the past decade reversed shoulder arthroplasty (RSA) has become more popular for complex fractures of the proximal humerus [8]. This case report presents a fragile 90-year-old woman with multiple comorbidities who had a serious complication following surgery for a proximal humerus fracture.

## Case history

**Fig. 1:**
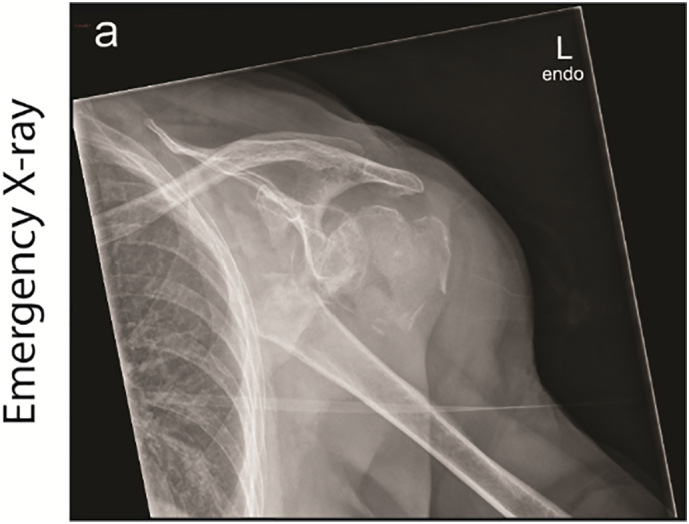
AP X-ray after trauma: Proximal humerus fracture with severe medial dislocation of the shaft, free greater tuberculum and multiple smaller osseous fragments.

A 90-year-old woman with a medical history of COPD, bilateral pulmonary embolism, diabetes and an anteroseptal infarction was brought to the emergency department after she fell in her apartment. On physical examination the left shoulder was very painful and her left leg was shortened and in exorotation. X-rays of the shoulder revealed a comminuted proximal humerus fracture with severe dislocation (Fig. 1). The pelvic radiograph showed a dislocated femoral neck fracture. No further abnormalities were detected after completing full trauma screening.

**Fig. 2:**
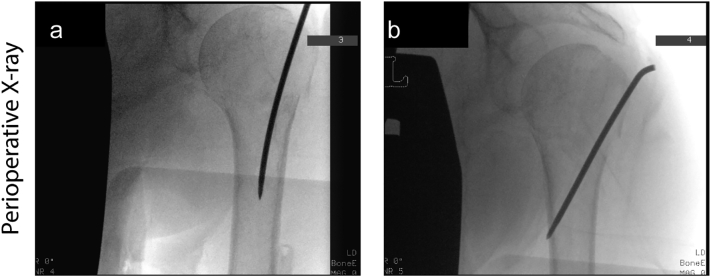
Intraoperative X-rays after closed reduction and percutaneous fixation with a single threaded K-wire.

A hemiarthroplasty was performed to treat the fractured hip. Multiple options were discussed as treatment for the proximal humerus fracture. Shoulder arthroplasty was considered, but due to the fragile status of the patient this was found to be too dangerous to perform in one go with the hemiarthroplasty of the hip. However, because of agonizing pain of the fractured left shoulder a closed reduction and percutaneous fixation with a threaded K-wire with bend-free end was performed during the surgery of the left hip to reduce pain and prevent further dislocation (Fig. 2).

**Fig. 3:**
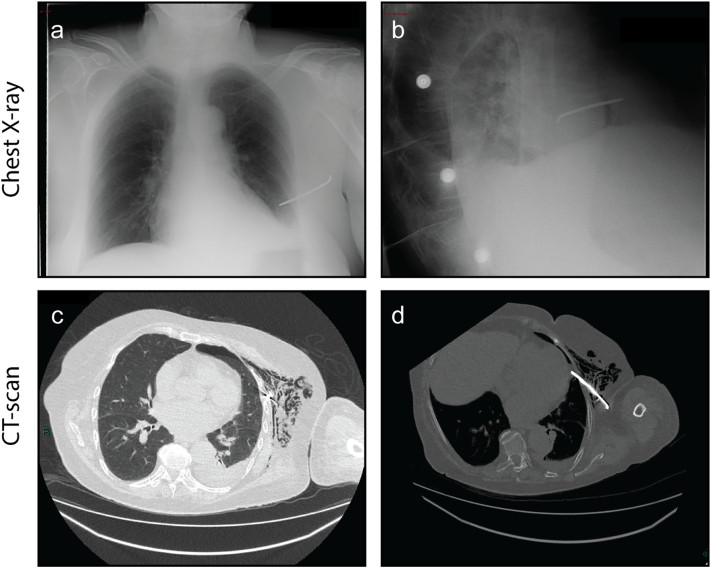
A–B: AP and lateral X-thorax. C: CT-thorax in pulmonary setting, ventrally a mild pneumothorax with subcutaneous emphysema. D: CT-thorax in bone setting, the K-wire located intrathoracically.

Postoperatively the patient recovered well at first. On postop day 3 the patient experienced increased pain in the left shoulder. A new X-ray showed dislocation and loss of repositioning. We decided that watchful waiting was the best option. On day 5 the patient experienced dyspnea. An X-thorax was performed which showed a migrated K-wire with a possible intrapulmonary tip (Fig. 3). To determine the exact location and involvement of the lung a CT was requested. The migrated K-wire was located in the lingula of the left lung. Secondarily there was a mild ventral pneumothorax with subcutaneous emphysema. The tip of the K-wire was situated near the pericardium; no pericardial fluid was visible (Fig. 3).

**Fig. 4:**
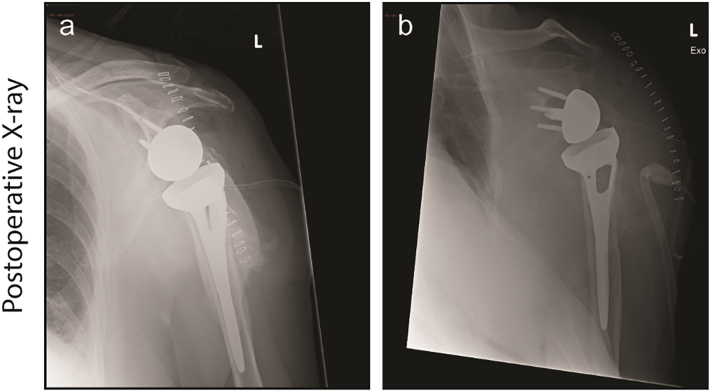
Control X-rays of the left shoulder one day after RSA: Free greater tuberculum, formation of callus and free osseous fragment laterally from the humeral shaft.

After consultation with a thoracic surgeon, the K-wire was removed by video-assisted thoracic surgery (VATS). After removal the patient slowly recovered, although the left shoulder remained subsequently painful. Three months later, when the patient was fit for surgery, an RSA was performed (Fig. 4). Three months after surgery passive motion of the left shoulder was supple and pain-free, with an active abduction of 90°.

## Discussion

A wide variety of techniques are available to treat proximal humerus fractures. Treatment focuses on pain relief and functional outcome [4]. Complex fractures are more prone to worse outcome so surgery should be considered. Various alternative surgical techniques to fixate proximal humerus fractures are described below.

The use of locking plates for osteoporotic fractures has increased in recent decades. It is suggested the rigid fixation improves mechanical stability, therefore resulting in better outcome [9]. Plate fixation allows anatomical reduction by controlling the different fragments of the fracture, and is used in 2- and 3-part, and sometimes 4-part fractures [10]. Intramedullary mailing is mostly suited for surgical neck and 2-part proximal humerus fractures [7]. However, a 2015 study found it to be inferior for postoperative pain and loss of function compared to percutaneous wiring [7]. These results can be explained by the damage done to the rotator cuff during placement of the nail and by an uncovered end of the nail. Ensuring the nail is well-buried with bone reduces postoperative pain and helps prevent the need for nail removal [11]. Historically, hemiarthroplasty has been the treatment of choice for unreconstructible proximal humerus fractures. In recent years this changed in favor of total shoulder arthroplasty (TSP) and RSA [12]. Unlike hemiarthroplasty, RSA doesn't need the greater tuberosity to heal in order to achieve a successful result. Patients undergoing RSA achieved increased forward flexion but reduced external rotation compared to hemiarthroplasty. RSA is additionally associated with a higher rate of postoperative complications than hemiarthroplasty [13]. There is no difference in clinical outcome scores, forward flexion or risk of reoperation between acute RSA and delayed RSA [14].

Closed reduction and percutaneous fixation is a minimally invasive technique with the advantage of minimal soft tissue damage and reduced surgery time, blood loss and postoperative pain [11]. In a 2015 study Tamimi et al. found a superior outcome in mean constant score in elderly patients with 2-part proximal humerus fractures [7]. Nevertheless, K-wire fixation is associated with poor reduction, malunion, pintract infection and pin migration [7,11]. It might nonetheless be a suitable option for low-maintenance patients with multiple comorbidities who aren't fit for major surgery [15]. Though it is uncommon, multiple reports have been written about K-wire migration into the thorax after shoulder surgery [16]. The exact mechanism remains unclear but theories suggest that the great freedom of movement of the shoulder, gravitational forces, muscle action, negative intrathoracic pressures during respiration, resorption of bone and osteoporotic bone may play a role [16]. Threaded tips of the K-wires with a bend-free end should be used to reduce the risk of migration [16]. The use of multiple K-wires increases the stability of the reduction [6]. We also advise not to bury the K-wire under the skin for postoperative visual control. Percutaneous K-wire fixation used to be a widely used technique for proximal humerus fractures until the introduction of more recent techniques such as arthroplasty [4,7,11]. In our case the isolated fracture of the humerus could have been treated well with RSA. Due to the fragile status of the patient and the fracture of the left hip we decided to fixate the proximal humerus fracture with a K-wire during surgery of the left hip.

## Conclusion

Proximal humerus fractures are common in the elderly population. Surgery should be considered for complex fractures to increase functional outcome. Depending on type of fracture and patient characteristics ORIF, intramedullary nailing or arthroplasty can be considered. Although percutaneous fixation is not the first choice for proximal humerus fractures, it can be a suitable treatment for low-maintenance and fragile patients. Multiple threaded K-wires with a unburied bend-free end should be used to reduce the risk of loss of repositioning or migration of the K-wire. In our case the repositioning seemed stable after drilling one threaded K-wire with a bent end. The use of multiple K-wires could have prevented migration by increasing stability. This case is an illustration and warning for trauma and orthopaedic surgeons who are considering use of percutaneous K-wire fixation in proximal humerus fractures.

## Authors' contributions

Writing of the report and literature research was done by AH and JH. MH was the operating surgeon who experienced the complication. Supervision of the project was done by JR and MH. All authors read and approved the final manuscript.

## Consent for publication

Written informed consent was obtained from the patient for publication of this case report. A copy of informed consent is available for review by the author.

## Declaration of competing interest

The authors have no conflicts of interests to declare.
